# Additional Brain Functional Network in Adults with Attention-Deficit/Hyperactivity Disorder: A Phase Synchrony Analysis

**DOI:** 10.1371/journal.pone.0054516

**Published:** 2013-01-31

**Authors:** Dongchuan Yu

**Affiliations:** 1 Key Laboratory of Child Development and Learning Science of Ministry of Education, Southeast University, Nanjing, Jiangsu, China; 2 Research Center for Learning Science, Southeast University, Nanjing, Jiangsu, China; Technical University of Madrid, Italy

## Abstract

We develop a method to construct a new type of functional networks by the usage of phase synchrony degree that is different from the widely used Pearson's correlation approach. By a series of very strict statistical tests, we found that there is an additional network in attention-deficit/hyperactivity disorder (ADHD) subjects, superimposing the original (normal) brain functional network corresponding to healthy controls. The additional network leads to the increase in clustering coefficient, cost, local efficiency, and global efficiency. Our findings are inconsistent with many previous researches (using the Pearson's correlation approach) revealing both increased and decreased functional connections between brain regions and many reports revealing that the brain functional networks of ADHD patients have slow information flow and low global efficiency. We also confirm that the additional network in ADHD subjects contains 6 communities, and three of them are associated with emotional control, sensory information integration, and motor control, respectively. Furthermore, we find that there is a pathway connecting the left insula and left anterior cingular gyrus via the frontal gyrus and putamen in the additional network in ADHD subjects. This implies that due to the pathway connecting brain regions in the salience network, the ADHD patients are more sensitive to external stimuli or internal thoughts and are easier to switch to the executive network and hence harder to inhibit. For clinical diagnostic purposes, we apply the 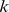-means clustering method to distinguish ADHD patients with healthy controls at the individual subject level, and obtain a meaningful diagnostic result. More interestingly, we find that the suggested technique using phase synchrony degree to construct functional networks may obtain higher classification accuracy than the method using the Pearson's correlation coefficient.

## Introduction

Attention-deficit/hyperactivity disorder (ADHD) [1]–[11] is characterized by developmentally inappropriate symptoms of excessive inattention, impulsivity, and motor restlessness. It is the most commonly studied and diagnosed psychiatric disorder in children, and affects approximately 5% of school-age children and frequently persists into adulthood [1]–[4]. The uncovering of the underlying mechanism, diagnosis and prediction of ADHD are of great importance but challenging problems in biomedical sciences.

Functional networks (FWs) [12] are widely used to quantify brain activity from experimental functional magnetic resonance imaging (fMRI) time series, and have been applied to study pathophysiology, medication effects, and potential tests to aid clinical diagnosis of ADHD. The ADHD pathophysiology associated with regional functional abnormalities has been uncovered in a very widespread area, including frontal, parietal, temporal, occipital, and subcortical regions [4], [13], [14]. Typical example includes anterior cingulate cortex (ACC), dorsolateral prefrontal cortex (DLPFC), ventrolateral prefrontal cortex (VLPFC), caudate, and putamen, among others. Although functional abnormalities [4], [13], [14] associated with ADHD have been studied extensively, it has not been fully understood the organization and community structure of FWs in the whole brain of ADHD patients.

Mathematically, FWs can be characterized by complex networks which are composed of nodes denoting voxels or brain regions that are linked by edges representing functional connections between nodes [12], [15]. Recent advances [12] in complex networks have been rapidly translated to studies of brain functional network organization that may be quantified by the topological structural parameters. Typical parameters include clustering coefficient, degree distribution, path length, efficiency, connection density, centrality, motifs, and community structure (see Refs. [12], [16] for a brief review). In particular, Girvan and Newman [17] provided the general notion of community structure in complex networks based on the fact that the nodes in networks are often found to cluster into tightly-knit groups (called communities) with a high density of within-group edges and a lower density of between-group edges. Neuroscientists have relished the possibility of using topological properties (or structural parameters) for the quantitative analysis of FWs in ADHD from fMRI data, but only recent reports [7], [18]–[21] using the concept of small-world properties [22] really made a major progress towards the understanding of the organization of FWs in the whole brain of ADHD patients. Furthermore, almost all researches [4], [6]–[11] focused on only the change of some particular linkages, pathways or even the organization of the whole brain networks, but no effort has been given to the organization of the different (functional) networks between the normal and disorder groups.

It should be remarked that the definition of functional connections in previous FW analysis methods [4], [6]–[11] is basically based on the Pearson's correlation approach (two signals are correlated if we can predict the variations of one as a function of the other). If the value of Pearson's correlation coefficient between two time-series exceeds a threshold, then there exists a linkage between them; otherwise, there is no connection between them. However, how to determine suitable thresholds is still an open problem [6]–[11], [15], [23].

On the other hand, phase synchrony (PS) [24] is originally defined as the locking of the phases of the coupled systems whereas their amplitudes may vary, and thus has more advantages than the Pearson's correlation approach (focusing on the linear prediction relation between two signals and being influenced by the amplitudes of signals) in understanding complex collective dynamical behavior. More importantly, many direct evidences supporting PS as a basic mechanism for brain integration have been extensively reviewed (see Ref. [25] for detailed information). Therefore, PS seems to be a promising tool for understanding the mechanisms of brain activities. Thus far, however, no effort has been given to apply PS to understand the organization of FWs in the whole brain in patients with brain disorder.

In this paper, we develop a method to construct a new type of FW from regional fMRI time series, in which PS degree [24], [25] between two regional fMRI time series is taken as the functional connection strength. For two kinds of fMRI time-series (corresponding to ADHD participants and healthy controls, respectively), we first calculate the FWs of each group, and then detect whether the organization of brain functional networks has been altered in patients with ADHD. Our method contains the following steps. First, we measure the resting-state fMRI imaging data of 21 ADHD participants (with 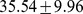 years) and 27 healthy controls (with 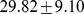 years). Then, by a series of typical operations with SPM8 (http://www.fil.ion.ucl.ac.uk/spm), we segment the resting-state fMRI imaging data into 116 regions using an anatomically labelled template (see Table 1 for detailed information) for each subject, where the cerebra is divided into 90 regions (45 in each hemisphere) and the cerebella into 26 regions (9 in each cerebellar hemisphere and 8 in the vermis). In this way, for each subject, we obtain 116 time-series for PS analysis, where each time-series characterizes the collective dynamics of a brain region. Next, we calculate the time-averaged PS degree between any two time series for each subject, and thus get 48 FW matrices with dimension 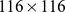. After that, by a group-averaged statistical method, we perform two one-tailed t-tests to determine whether the mean of each element of FW matrices within ADHD subjects is different from that within healthy group. In this way, we may in principle get two group-averaged statistical difference networks 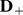 and 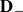. We then detect whether there are some community structure in networks 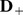 and 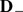 by the usage of the concept of modularity [17]. Once some community structure has been identified in networks 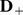 and 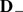, we may quantify further the topological roles [26] of each node in terms of its density of intra- and inter-community connections, and thus assign the roles of 116 brain regions and interpret some of them by the usage of their widely-accepted neurophysiological functions.

**Table 1:** Cortical and subcortical regions defined in the anatomically labelled template.

Index	Region name	Abbr.	Index	Region name	Abbr.
1,2	Precental gyrus	PreCG	63,64	Supramarginal gyrus	SMG
3,4	Superior frontal gyrus, dorsolateral	SFGdor	65,66	Angular gyrus	ANG
5,6	Superior frontal gyrus, orbital part	ORBsup	67,68	Precuneus	PCUN
7,8	Middle frontal gyrus	MFG	69,70	Paracentral lobule	PCL
9,10	Middle frontal gyrus, orbital part	ORBmid	71,72	Caudate nucleus	CAU
11,12	Inferior frontal gyrus, opercular part	IFGoperc	73,74	Lenticular nucleus, putamen	PUT
13,14	Inferior frontal gyrus, triangular part	FGtriang	75,76	Lenticular nucleus, pallidum	PAL
15,16	Inferior frontal gyrus, orbital part	ORBinf	77,78	Thalamus	THA
17,18	Rolandic operculum	ROL	79,80	Heschl gyrus	HES
19,20	Supplementary motor area	SMA	81,82	Superior temporal gyrus	STG
21,22	Olfactory cortex	OLF	83,84	Temporal pole: superior temporal gyrus	TPOsup
23,24	Superior frontal gyrus, medial	SFGmed	85,86	Middle temporal gyrus	MTG
25,26	Superior frontal gyrus, medial orbital	ORBsupmed	87,88	Temporal pole: middle temporal gyrus	TPOmid
27,28	Gyrus rectus	REC	89,90	Inferior temporal gyrus	ITG
29,30	Insula	Ins	91,92	Cerebelum_Crus1	CERcr1
31,32	Anterior cingulate and paracingulate gyri	ACG	93,94	Cerebelum_Crus2	CERcr2
33,34	Median cingulate and paracingulate gyri	DCG	95,96	Cerebelum_3	CER3
35,36	Posterior cingulate gyrus	PCG	97,98	Cerebelum_4_5	CER4_5
37,38	Hippocampus	HIP	99,100	Cerebelum_6	CER6
39,40	Parahippocampal gyrus	PHG	101,102	Cerebelum_7	CER7
41,42	Amygdala	AMYG	103,104	Cerebelum_8	CER8
43,44	Calcarine fissure and surrounding cortex	CAL	105,106	Cerebelum_9	CER9
45,46	Cuneus	CUN	107,108	Cerebelum_10	CER10
47,48	Lingual gyrus	LING	109	Vermis_1_2	Ver1_2
49,50	Superior occipital gyrus	SOG	110	Vermis_3	Ver_3
51,52	Middle occipital gyrus	MOG	111	Vermis_4_5	Ver4_5
53,54	Inferior occipital gyrus	IOG	112	Vermis_6	Ver6
55,56	Fusiform gyrus	FFG	113	Vermis_7	Ver7
57,58	Postcentral gyrus	PoCG	114	Vermis_8	Ver8
59,60	Superior parietal gyrus	SPG	115	Vermis_9	Ver9
61,62	Inferior parietal, but supramarginal				
	and angular gyri	IPL	116	Vermis_10	Ver10

For clinical diagnostic purposes, we finally apply the 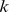-means clustering method to distinguish ADHD patients with healthy controls at individual subject level, in which the community structure measure has been used as the feature parameter. We also compare the difference of classification accuracy between two cases, i.e., the suggested technique using phase synchrony degree to construct functional networks and the method using the Pearson's correlation coefficient.

## Materials and Methods

### Participants and original imaging data

The subjects involved in this study include 21 adult participants with ADHD (with 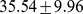 years) and 27 healthy volunteers (with 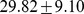 years). Each participant underwent one resting state scan consisting of 197 contiguous whole-brain functional volumes using echo planar imaging on a Siemens 3.0 Tesla Allegra (TR = 2000 ms; TE = 25 ms; flip angle = 90, 39 slices, matrix = 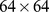; FOV = 192 mm; acquisition voxel size = 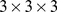 mm). The original resting-state fMRI imaging data of all participants can be downloaded from the open-access website http://www.nitrc.org/projects/fcon_1000/.

### Imaging data processing

We carried out the imaging data preprocessing using SPM8 (http://www.fil.ion.ucl.ac.uk/spm). For each subject, we first performed slice timing to correct the differences in acquisition time between slices during sequential imaging, and then realigned all EPI functional volumes to reduce the head-motion correction. Next, we spatially normalized to transform all T1-weighted volumes to MNI152 standard brain space. We further performed spatial registration to map all EPI functional volumes to individual T1-weighted image with spatial resolution of 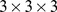 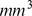. After that, we removed the linear trends, performed the spatial smoothing with an 8 mm full-width half maximum (FWHM) Gaussian kernel, and performed a temporal band-pass filtering (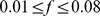 Hz) to reduce the effects of low-frequency drift and high-frequency noise. Finally, we extracted 116 regional time-series from filtered preprocessed resting state data using the automated anatomical labeling (see Table 1 for detailed information), where the cerebra is divided into 90 regions (45 in each hemisphere) and the cerebella into 26 regions (9 in each cerebellar hemisphere and 8 in the vermis). In this way, for each subject, we have obtained 116 time-series for further analysis, where each time-series characterizes the neural activity of a brain region.

### Phase synchrony

By the usage of Hilbert transform, the instantaneous phase [24], [25] 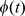 of a real signal 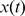 can be defined as.
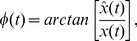
(1)where 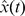 is the Hilbert transform of 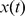 and is given by.



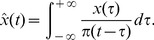
(2)The phase synchrony [24], [25] between two time-series 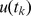 and 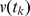 is satisfied if.
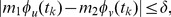
(3)where 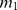 and 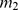 are integers (typically 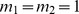), 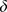 is a small positive, and 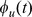 and 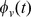 are the instantaneous phase of 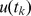 and 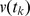, respectively. The above definition is the original version of phase synchrony, and has been widely used in the physics society.

In the current study, we use a time-averaged phase synchrony degree to characterize the strength of functional connection between two brain regions 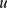 and 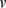, which is given by
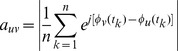
(4)where 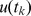 and 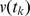 are fMRI time-series representing the neural activity of brain regions 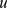 and 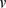, respectively; and 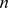 is the length of time series. It is clear that 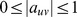. The closer the value of 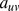 is to its theoretical maximum 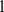, the stronger the phase synchrony between brain regions 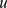 and 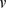.

### Functional networks

For any two different brain regions 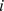 and 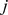, we determine the strength of functional connection between them according to Eq. (4). Here we do not deal with the self-synchrony case and thus set 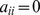 for all 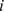. In this way, we obtain a whole-brain FW characterizing by 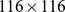 matrix 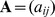 for each subject. It is clear that matrix 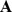 is weighted and symmetry, with zero diagonal elements.

We calculate the FW of all 48 subjects (including 21 ADHD and 27 healthy participants). For better presentation, we denote the FW of ADHD subjects and that of healthy group by 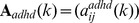 and 
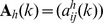
, respectively, where 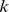 represents the series number of subjects.

### Statistical difference networks

We apply a group-averaged statistical method to identify the difference between FWs in ADHD and that in healthy group. For this purpose, let us denote 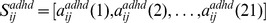 and 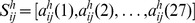. For each 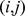 pair, we perform two one-tailed t-tests to determine whether the mean of series 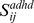 is greater than that of 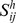, as well as to detect whether the mean of series 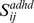 is less than that of 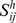. In this way, we may in principle get two unweighted matrices: 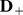, corresponding to pairs 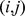 satisfying the mean of series 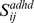 greater than that of 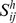 statistically; and 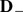, corresponding to the pairs 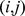 satisfying the mean of series 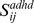 less than that of 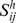 statistically.

### Community structure detection

It is of interest to detect whether there exists some community structure in a network. For this purpose, we use a widely-used measure of the community structure given by:
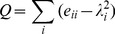
(5)where 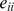 are the fraction of links that connect two nodes inside the community 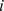, 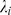 the fraction of links that have one or both vertices inside of the community 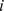, and the sum extends to all communities 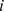 in a given network. The closer the value of 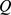 is to its theoretical maximum 1, the stronger the community structure.

To determine the community structure, we in practice applied the famous modularity maximization algorithm, which detects communities by searching the maximum value of 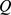 over possible divisions of a network. The value of 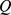 other than 0 indicates a deviation from randomness, and in practice that greater than about 0.3 appears to indicate significant community structure [17].

### Topological roles of nodes in networks with community structure

Once community structure in a network has been identified, we may quantify further the topological roles of each node in terms of its density of intra- and inter-community connections. Our method basically stems from the concept of node roles used for the quantitative analysis of the worldwide air transportation network [26], but we make some modifications such that the measure of topological roles is more suitable for whole brain functional networks constructing from fMRI data.

We measure the intra-community connectivity of node 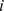 by its degree z-score within the 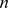-th community, given by
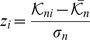
(6)where 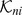 is the number of edges connecting node 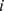 to other nodes in the 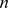-th community, 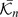 is the average of 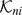 over all nodes in the 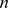-th community, and 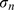 is the standard deviation of the intra-community degrees in the 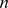-th community. This within-community degree z-score measures how “well-connected” node 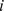 is to other nodes in the community.

We further measure the inter-community connectivity of node 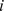 in the 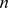-th community by
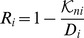
(7)where 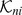 is the number of edges connecting node 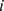 to other nodes in the 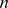-th community, and 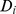 the degree of node 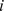 in the whole network. The value of 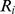 close to 0 indicates that node 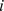 basically connects to all other nodes in the same community, and that close to 1 indicates that node 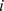 basically connects to nodes in other communities. It should be remarked that the definition of inter-community connectivity is different from that used in the work [26] for the worldwide air transportation network.

The role of each node can be assigned by the values of 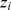 and 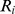. For instance, the node with bigger 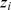 and 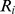 will have higher within-degree and more links connected to other communities, and thus be more important to the information flow processing of the whole network. Therefore, depending on the values of 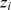 and 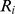, we may classify all nodes into six classes: (T1) “backbone hubs” satisfying 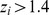 and 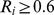, (T2) “transfer hubs” satisfying 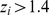 and 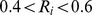, (T3) “local hubs” satisfying 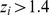 and 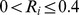, (T4) “backbone non-hubs” satisfying 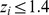 and 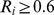, (T5) “transfer non-hubs” satisfying 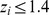 and 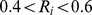, and (T6) “local non-hubs” satisfying 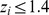 and 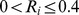.

### Binarization of weighted FWs

For a given threshold 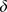, a weighted FW 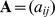 may be binarized as an unweighted FW 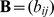 given by:
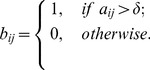
(8)


It is clear that the bigger the value of 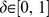 the sparser the matrix 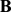.

### Classification of ADHD and healthy subjects using 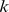-means clustering

It should be remarked that the above group-averaged method is inadequate to assist in clinical diagnostic purposes, since clinical diagnostic decision making requires the ability to reliably distinguish normal from abnormal at the individual subject level. To cope with this drawback of the group-averaged method, we develop a classification method with 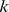-means clustering to distinguish ADHD patients with healthy controls at individual subject level, where the feature parameter used for classification is the value of 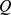 given by Eq. (5). 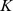-means clustering is a widely used method of cluster analysis which aims to partition 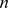 observations into 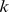 clusters in which each observation belongs to the cluster with the nearest mean. The basic idea of 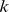-means clustering is briefly described in the following.

Given a set of measures 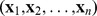, with each measure being a 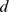-dimensional vector, 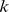-means clustering aims to partition the 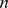 measures into 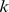 (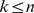) clusters 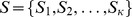 so as to minimize the following variable 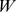:
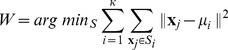
(9)where 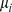 is the mean of points in cluster 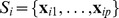, and the true 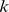 clusters are 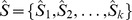 with 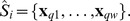


The solution of this optimal problem is associated with an iterative processing. For a 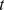-th iterative estimate of means 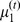, 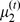, 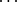, 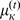, we assign each measure to the cluster with the closest mean by the following rule:
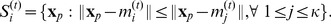
(10)


We then obtain a updated estimate of means by
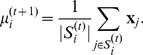
(11)


In practice, we first randomly choose 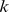 measures from the data set and take them as the initial estimate of means, then obtain an updated estimate by Eqs. (10) and (11), and finally complete the iterative operation when the assignments no longer change.

For any element 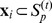 with 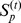 being the estimated 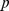
th cluster when the above iterative operation is stopped (i.e., the assignments no longer change), it can be classified correctly if 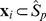 with 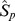 being the true 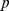
th cluster. Therefore, the classification accuracy rate using 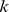-means clustering is determined by the value 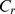 given by
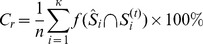
(12)where 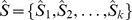 and 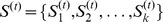 denote the true classification and the estimated one using 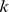-means clustering, respectively; 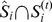 is the intersection of sets 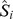 and 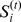; and 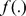 denotes the operation to calculate how many elements in a set. It is clear that 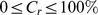. The closer the value of 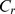 is to its maximum 100%, the higher the classification accuracy rate.

It should be remarked that 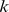-means clustering may achieve a clustering result even when the estimated number of clusters is different from the true (expected) one. So we usually need a priori knowledge associated with the true number of clusters to achieve an acceptable result using 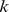-means clustering.

## Results

### Statistical difference networks

We interestingly found that the hypothesis, that the mean of series 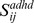 is less than that of 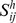, is null for any 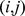 (one-tailed t-test, 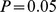). This implies that PS in ADHD subjects is statistically enhanced, compared with that in healthy group.

**Figure 1:**
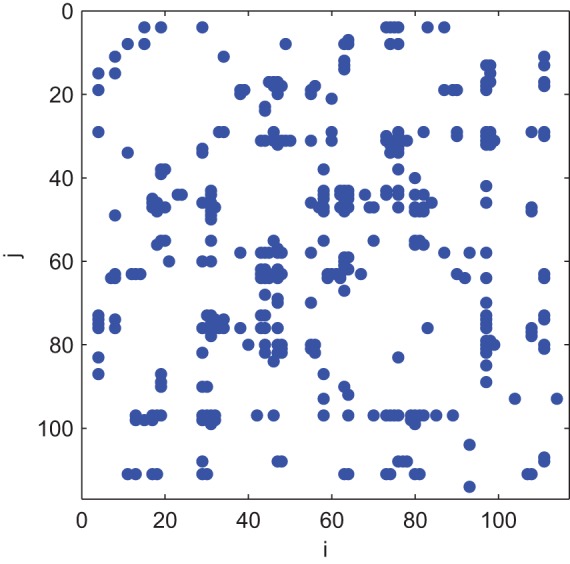
Map of the difference network 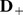, where the site 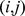 plotting blue point indicates 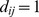, and otherwise 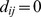.

**Figure 2:**
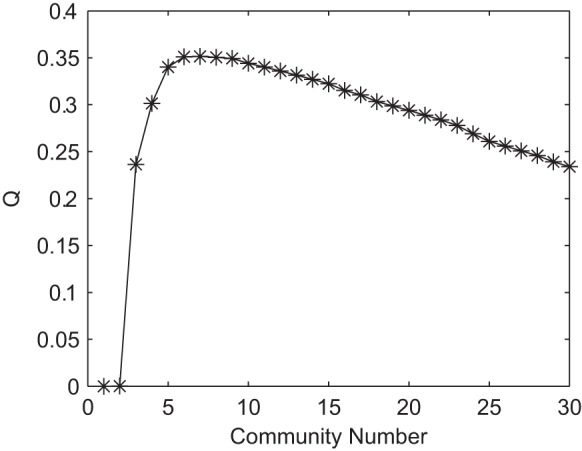
The community structure detection processing is shown. The optimal processing of 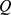 over all possible divisions is plotted, where 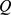 achieves its maximum when the network is clustered into 6 communities.

**Figure 3:**
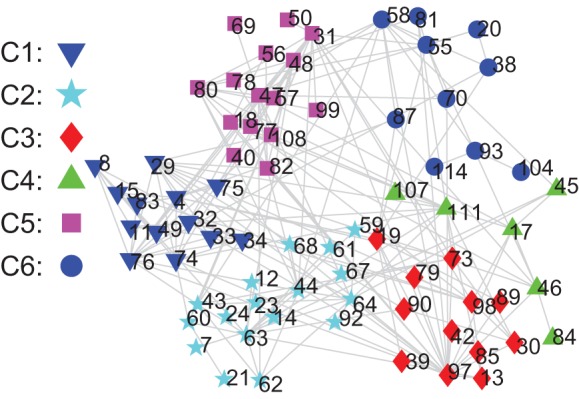
Community structure in the difference network 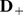 is sketched. Six communities are found in the difference network 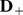, where the nodes in communities 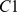
–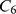 are plotted with 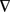, 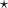, ⧫, 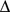, 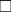, and 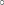, respectively.

**Figure 4:**
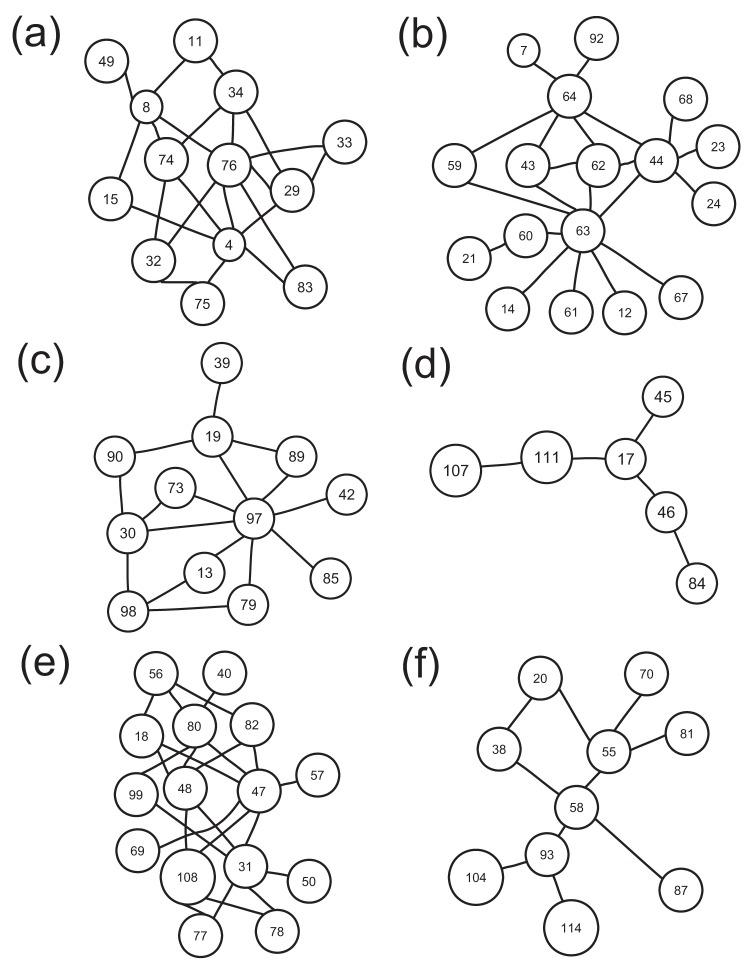
The connections within each community are plotted. The connections within each community (from C1 to C6) are sketched by Panels (a)–(f), respectively.

Figure 1 shows the statistical difference network 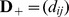 between ADHD and healthy group, where 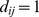 if the mean of series 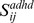 is greater than that of 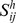 (one-tailed t-test, 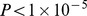). It is of interest to detect if there is some community structure in the network 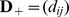. Figure 2 summarizes our results and shows the maximal value of 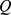 over all possible divisions for a given number of communities. It is clear that the maximum of value 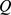 greater than 0.3 is achieved when the network is divided into 6 communities. This indicates that the network 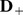 can be clustered into 6 significant communities as shown in Fig. 3, where the connections within each community are sketched in Fig. 4.

We interestingly found from Fig. 4 that: i) The first community (C1) is associated with emotional control, mainly including insular (node 29), frontal gyrus (nodes 4, 8, 11, 15), cingulate (nodes 32, 33, 34), and lenticular nucleus (nodes 74, 75, 76); ii) The second community (C2) contains most brain regions (nodes 59, 60, 61, 62, 63, 64, 67, 68) in parietal lobe that integrates sensory information from different modalities, particularly determining spatial sense and navigation; and iii) The supplementary motor area (node 19), insular (node 30), parahippocampal gyrus (node 39), amygdala (node 42), putamen (node 73), and cerebelum (nodes 97, 98) are clustered into the third community (C3), associated with motor control. That is, three meaningful communities (i.e., C1, C2, C3), associated with ADHD pathology, have been identified.

We hypothesize that some intra-community connections might lead to the occurrence of ADHD. Actually, some intra-community connections in ADHD subjects have been reported, such as the connection between ACG and pallidum, but some of them has not been shown before, such as the functional links related to the caudate and posterior cingulate gyrus.

**Figure 5:**
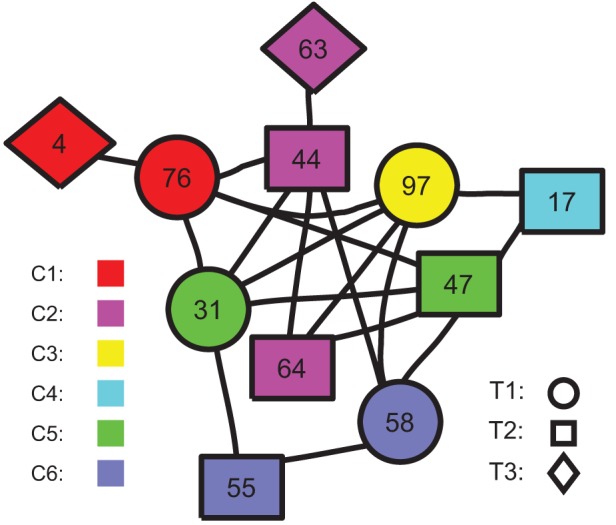
The intra-connections between T1, T2, and T3 nodes in all communities.

It is of interest to quantify further the topological roles of nodes in the difference network 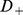, in terms of its density of intra- and inter- community connections. By the measure of 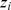 and 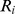 given by Eqs. (6) and (7), all nodes in 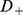 can be grouped into six classes: T1 (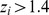, 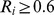), T2 (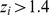, 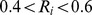), T3 (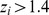, 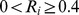), T4 (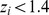, 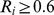), T5 (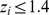, 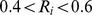), and T6 (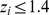, 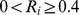). Table 2 summarizes our results and lists the detailed node classification for each community. Figure 5 shows the intra-connection topology between T1, T2, and T3 nodes in all communities (C1-C6).

**Table 2:** Community detection of the difference network 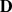.

#	T1	T2	T3	T4	T5	T6
C1		76	4	29	32, 49, 74, 75	8, 11, 15, 33, 34, 83
C2		44, 64	63		43, 60	7, 12, 14, 21, 23, 24, 59, 61, 62, 67, 68, 92
C3	97			73, 98	13, 19, 90	30, 39, 42, 79, 85, 89
C4		17		45, 46, 111		84, 107
C5	31	47			18, 80, 82, 108	40, 48, 50, 56, 57, 69, 77, 78, 99
C6	58	55		70, 81, 87	38	20, 93, 104, 114

### Community structure change in adults with ADHD

**Figure 6:**
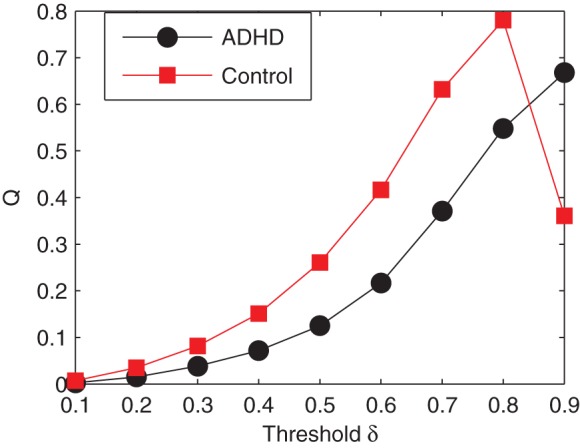
The group-average difference of 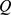 between ADHD patients and controls is changed with the binarization threshold 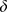. The group-average value of 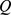 within ADHD patients (circled line) and that within controls (squared line) are plotted for each 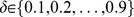.

For a given 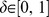, we may first achieve the binary FW 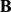 for each subject in terms of the rule (8), then calculate the value of 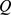 corresponding to the obtained 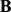 by Eq. (5), and finally average the value of 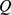 within ADHD group and that within healthy one, respectively. Figure 6 summarizes our results and shows the the difference of group-averaged value of 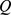 between ADHD and healthy group for 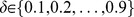. As shown in Fig. 6, the group-averaged value of 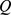 within ADHD group is less than that within healthy group for 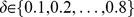. We found that the binary FWs of healthy controls in the case of 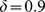 become very sparse and partial of them even null matrices. This directly results in that the group-averaged value of 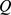 within ADHD group is bigger than that within healthy group for 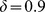, as shown in Fig. 6.

We may conclude from Fig. 6 that healthy controls have stronger community structure compared with ADHD subjects. This, in combination with the fact that the difference network between ADHD and healthy group has community structure, implies that the ADHD subjects may have an additional community structure, superposing the original (normal) community structure in healthy controls. The additional community structure plays a crucial role for the ADHD pathology, and thus the understanding of its role might be a new direction of ADHD research.

### Classification using 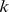-means clustering

The above group-averaged methods provide some new insights for the understanding of ADHD pathology, but they are inadequate to assist in clinical diagnostic purposes since clinical diagnostic decision making requires the ability to reliably distinguish normal from abnormal at the individual subject level. To cope with this drawback of the group-averaged methods, we develop a classification method with 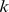-means clustering to distinguish ADHD patients with healthy controls at individual subject level.

**Figure 7:**
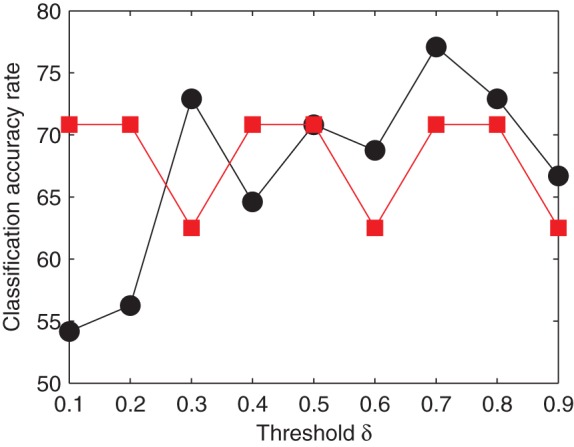
The classification accuracy rate being calculated by two different methods (i.e., our suggested technique using phase synchrony and the counterpart using Pearson's correlation) are compared. For each 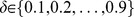, the accuracy rate of classifying each ADHD or control subject into the correct group is calculated using 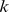-mean clustering for two cases: (A) functional networks constructing by phase synchrony (black points); and (B) functional networks constructing by Pearson's correlation approach (red squares), where 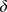 is the binarization threshold and the community measure 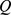 is taken as the feature parameter for 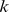-mean clustering.

For a given 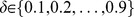, we calculate the value of 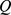 corresponding to the binary matrix 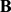 given by Eq. (8) for each subject. As a result, we obtain 48 measures of 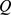 for all subjects for each 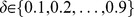. Then, for each 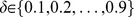, we partition 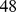 measures of 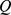 into 2 clusters by 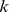-means clustering method. Finally, according to Eq. (12), we calculate the classification accuracy rate 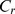 for each 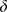. Figure 7 summarizes our results and shows the classification accuracy rate (black points) for each 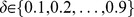. It is clear that the classification accuracy rate has its maximum 77.08% (implying that the classification error rate is 22.92%) when 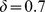. In addition, our method constructing functional networks by PS (black points) obtains higher classification accuracy rate than that by Pearson's correlation approach (red squares) when 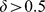, where the maximal classification accuracy rate of the latter is 70.83%. This implies that PS seems to be a promising tool for understanding the organization of FWs in the whole brain in patients with brain disorder. It should be remarked that the binarization of weighted FWs using PS method also suffers from the problem of threshold, similarly as that using Pearson's correlation approach. Even so, our results (see Fig. 7) suggest that 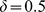 seems to be the critical value to show the advantage of our method using PS, and thus 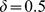 might be the minimal threshold for the binarization of weighted FWs using PS. On the other hand, the classification accuracy rate has its maximum when 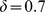. This seems to imply that the maximal threshold for the binarization of weighted FWs might be 0.7. Therefore we reasonably hypothesize that the threshold for the binarization of weighted FWs using PS might range from 0.5 to 0.7 for ADHD patients. We are now seeking more experimental evidences to support such a hypothesis.

## Discussion

We develop a method to construct a new type of functional networks by the usage of phase synchrony degree that is different from the widely used Pearson's correlation approach. This method has proven to be very informative and has been successfully applied to identify altered community structure and functional circuits in ADHD subjects. Our suggested technique to construct functional networks, in combination with 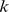-means clustering method, may further be used to distinguish ADHD patients with healthy controls at individual subject level, and more importantly, may obtain higher classification accuracy than the method using the Pearson's correlation, as illustrated in Fig. 7. This is consistent with other evidences [25] supporting phase synchrony as a basic mechanism for brain integration. Therefore, phase synchrony seems to be a promising tool for understanding the mechanisms of brain activities.

Furthermore, by a series of very strict statistical tests, we found surprisingly that phase synchrony degree between any two brain regions in ADHD subjects is statistically enhanced, compared to that in healthy group. That is, there is an additional network in ADHD subjects, superposing the original (normal) brain functional network corresponding to healthy controls. This is inconsistent with many previous researches (using the Pearson's correlation approach) revealing both increased and decreased functional connections between brain regions.

**Figure 8:**
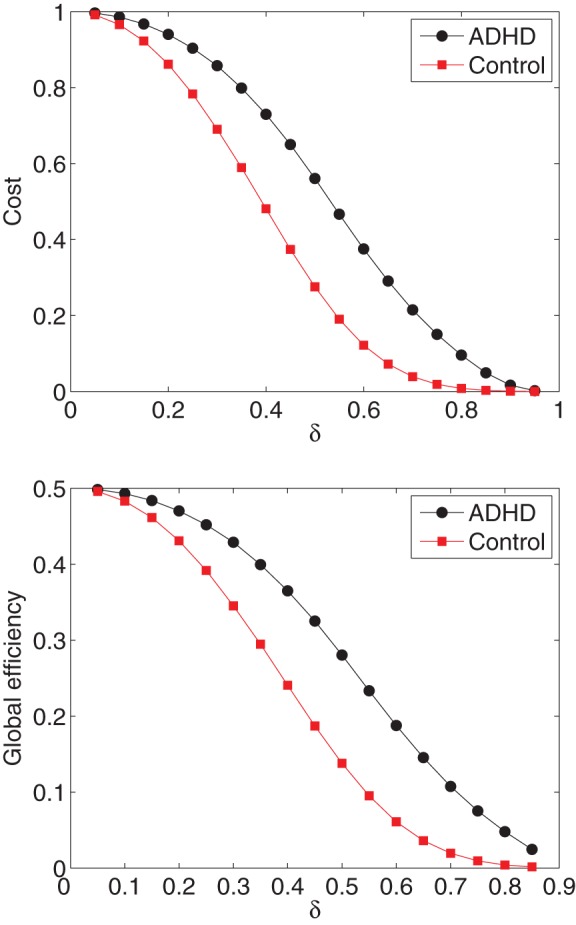
The group-average difference of cost and global efficiency between ADHD patients and controls is changed with the binarization threshold 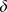. (Top) The group-average value of cost within ADHD patients (circled line) and that within controls (squared line) are plotted for each 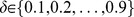. (Bottom) The group-average value of global efficiency within ADHD patients (circled line) and that within controls (squared line) are plotted for each 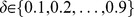.

The additional network in ADHD group leads to the increase in clustering coefficient, cost, local efficiency, and global efficiency, as illustrated in Fig. 8 where the values of cost and global efficiency of ADHD patients are greater than that of healthy controls. This is inconsistent with many previous studies (using the Pearson's correlation approach) revealing that the brain functional networks of ADHD patients have slow information flow and low global efficiency, compared to that of healthy controls. The increased local and global efficiency in ADHD group might be attributable to a functional reorganization (i.e., brain plasticity), leading to that the ADHD patients become easier to switch to executive network and thereby harder to inhibit. We thereby suspected that the increase of local and global efficiency in ADHD observed here might lead to a functional reorganization responsible for triggering the attention-deficit or hyperactivity disorder. A similar result has been reported by De Vico Fallani *et al*. [27] where they found an increased local efficiency in spinal cord injured patients that is attributable to a functional reorganization.

The additional network in ADHD subjects plays a crucial role for the ADHD pathology, and thereby it is of significance to analyze the organization and structure of the additional network at the system level. Community structure enables us to identify the sub-structures within a network, and provide us insight into how network function and topology affect each other. Several methods for community finding have been developed and employed with varying levels of success. Detailed discussion and comments can be found in Ref. [28]. Here we applied the modularity maximization algorithm that detects communities by searching over possible divisions of a network for one or more that have particularly high modularity. Since exhaustive search over all possible divisions is usually intractable, practical algorithms are based on approximate optimization methods such as greedy algorithms, simulated annealing, or spectral optimization, with different approaches offering different balances between speed and accuracy [29]. Here here we focused on the analysis of community structure of brain functional networks constructed by fMRI data, and found interestingly that the additional network in ADHD group contains 6 communities, and three of them (i.e., C1, C2, and C3) are associated with emotional control, sensory information integration, and motor control, respectively. In particular, the connections between those nodes with important roles in the additional network may be crucial for the ADHD pathology, as shown in Fig. 5 where the connections between T1, T2, and T3 nodes are plotted.

**Figure 9:**
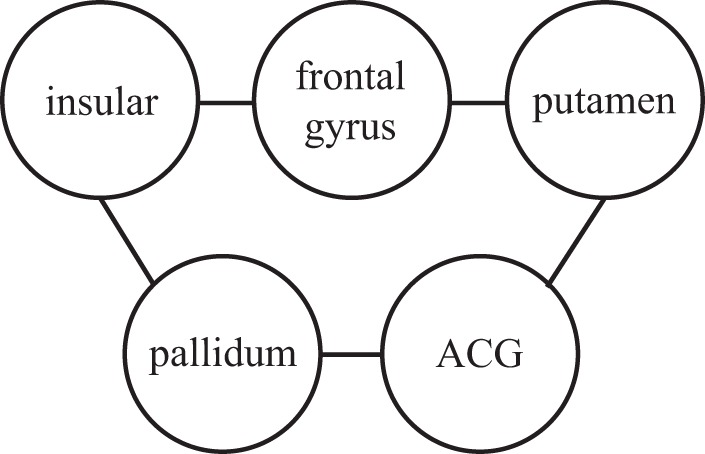
The circuit connects insular (node 29), frontal gyrus (nodes 4), putamen (nodes 73, 74), ACG (node 31), and pallidum (node 76).

The salience network [30], comprising insula and anterior cingular gyrus, is thought to play a role in recruiting relevant brain regions for the processing of sensory and emotional information. More precisely, the primary role of the salience network is the integration of sensations, internally generated thoughts and information about goals and plans in order to update expectations about the internal and external milieu and, if necessary, to allow action to be initiated or modified. The salience network serves as a dynamical switcher between internal status (default mode network) and central executive network [30]. Both states can be spontaneously activated and integrated by situational stimuli that, for the given individual, provide sufficient intrinsic satisfaction or threat to stimulate and sustain response [30]. We found interestingly (see Fig. 9) that there is a pathway connecting left insula (node 29) and left anterior cingular gyrus (node 31) via frontal gyrus (nodes 4) and putamen (nodes 73, 74) in the additional network in ADHD subjects. Our finding here suggests that, due to the pathway connecting brain regions in the salience network, the ADHD patients are more sensitive to external stimuli or internal thoughts, and are easier to switch to the executive network and hence harder to inhibit. Therefore, the pathway connecting brain regions in the salience network seems to be contributed to the ADHD pathology. This is the first time to identify a pathway linking brain regions in the salience network of ADHD patients. It should be remarked that the insula region, as the key role of the salience network, is the wellspring of social emotions, things like lust and disgust, pride and humiliation, guilt and atonement, and gives rise to moral intuition, empathy and the capacity to respond emotionally to music.

The amygdalae performs primary roles in the formation and storage of memories associated with emotional events, while the anterior cingular gyrus plays a critical role in up-dating the prediction models and has been shown to be involved in both social and reward related associative learning. We found interestingly that there is a pathway connecting the amygdala (node 42) and the anterior cingular gyrus (nodes 31, 32) via the cerebelum (nodes 97) in the additional network in ADHD subjects. Our finding here suggests that that the pathway connecting the amygdala and the anterior cingular gyrus might contribute to the ADHD pathology. This is inconsistent with previous results showing an decreased activities in the reward system in ADHD patients.

We also confirmed that some previously identified circuits associated with ADHD were found, such as the connection between ACG and pallidum, but some of them were not found to be altered in our current research, such as the functional links related to the caudate and posterior cingulate gyrus.

The group-averaged method provides some new insights for the understanding of ADHD pathology, but it is inadequate to assist in clinical diagnostic purposes since clinical diagnostic decision making requires the ability to reliably distinguish normal from abnormal at the individual subject level. For clinical diagnostic purposes, we applied the 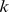-means clustering method to classify each ADHD or control subject into the correct group. As illustrated in Fig. 7 (black points), the classification accuracy rate has its maximum 77.08% (implying that the classification error rate is 22.92%). This implies that we have obtained a meaningful diagnostic result using the suggested techniques.

The suggested techniques are not limited to only the analysis of ADHD and can be easily extended to other kinds of brain disorders including (social) emotional or memory disorders. Indeed, we may provide a new method to construct functional networks by the usage of phase synchrony degree that is different from the Pearson's correlation approach, and hence measure the functional connectivity change at the pathway or network level. Our findings here focused on the resting-state fMRI data, and are helpful to identify the functional connectivity change to internal or external stimuli promoting behavioral responses.

We obtained the 116 regional time-series from filtered preprocessed resting state data using an automated anatomical labeling (AAL) template (see Table 1 for detailed information). Other available templates can also be applied to explore the effect of the templates on the networks architectures. It should be remarked that our findings here could be potentially influenced by both the “standard” operations using SPM8 and the definition of phase synchrony degree. Thus far, however, no systematic research has been conducted to evaluate the effects of the preprocessing approaches on the functional networks. Nevertheless, some scientists made efforts to the effect of frequency band on the functional networks. Achard et al. [31], for instance, showed that brain functional networks exhibited small-world properties at multiple time scales, but most salient in the low-frequency interval 0.03–0.06 Hz. To data, there are serval methods to define the phase of a complex signal using linear phase, Hilbert transform, or wavelet transform. Hence one could develop the corresponding functional networks depending on the different phase definition. In the future studies, it would be of interest to systematically analyze the effects of different preprocessing procedures on the topological properties of brain networks.
